# Cardiogenic shock in thyroid storm: A biventricular impella (Bi‐Pella) approach

**DOI:** 10.1002/ccr3.3657

**Published:** 2021-02-16

**Authors:** Evan Caruso, Elias Iliadis

**Affiliations:** ^1^ Department of Cardiology Cooper University Hospital Camden NJ USA

**Keywords:** biventricular failure, hemodynamics of right ventricular failure, mechanical circulatory support, thyrotoxic cardiomyopathy

## Abstract

The use of biventricular impella support in patients with acute, reversible causes of biventricular cardiogenic shock may play a role in shortening the time to recovery and preventing significant negative outcomes such as renal or hepatic failure.

## Graphical abstract

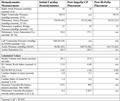

## 1 INTRODUCTION

### 1.1 History of presentation

A 31‐year‐old man with a history of hyperthyroidism presented to the emergency department with rapid atrial fibrillation with heart rates up to 200 bpm and was found to be in thyroid storm. Following the initiation of an esmolol drip, the patient became lethargic, cool to touch, and hypotensive requiring norepinephrine.

Laboratory data showed multi‐organ failure, and echocardiography revealed severe global hypokinesis with an EF of 5%. He was taken urgently for cardiac catheterization, which revealed a cardiac output of 2.8 L/min, a cardiac power output of 0.47, a pulmonary artery pulsatility index (PAPi) of 0.535, an RVSWI of 3.19, and an RA:PCWP ratio of 1.03 consistent with biventricular failure.

An impella CP was placed with improvement in left ventricular function at 30 minutes, but with a persistently low PAPi, low RVSWI, and an elevated RA:PCWP ratio consistent with severe right ventricular dysfunction. An RP impella was placed with improvement in biventricular function and a significant improvement in mental status.

His clinical status rapidly improved with mechanical support and treatment of his hyperthyroidism. He was weaned off of mechanical circulatory support within 3 days without any significant complications and was initiated on medical therapy for heart failure. On discharge, he had recovery of left ventricular ejection function to 35% and normal RV function. At his 6‐month follow‐up visit, he had normalization of biventricular function.

A 31‐year‐old man with a history of Grave's disease presented with 5 days of lower extremity swelling and shortness of breath. He had been on methimazole as an outpatient for several years, but had not taken it for several weeks due to loss of his insurance. On admission, he had an irregular heart rhythm with rates up to 200 bpm and a blood pressure of 110/70. An EKG was done showing rapid atrial fibrillation.

Laboratory data were consistent with thyroid storm. He was initiated on propranolol, PTU, and hydrocortisone with admission to the ICU. Heart rates continued to be elevated, and he was started on an esmolol drip. With rate control, he became confused, lethargic, cold, and hypotensive.

A bedside echocardiogram was performed showing 4 chamber dilatation and biventricular failure with an ejection fraction of 5%. Laboratory data showed developing shock (Table 1). He was started on norepinephrine and taken to the cath lab for invasive hemodynamic evaluation.

**TABLE 1:** Laboratory trend during hospitalization

Time	Admission (Hospital day 0)	Hospital day 1^a^	Hospital day 1^b^	Hospital day 2	Hospital day 3^c^	Hospital day 4^d^	Hospital day 5
Shock labs							
Lactate (mmol/L)	N/A	11.6	6.3	3.4	2	1.5	1.4
Creatinine (mg/dL)	0.48	0.8	1.25	1.42	1	0.75	0.4
Total Bili (mg/dL)	3.2	5.1	8.1	12.2	7.2	5.7	3.1
AST (U/L)	58	Hemolyzed	Hemolyzed	1807	609	416	123
ALT (U/L)	117	Hemolyzed	Hemolyzed	1324	1154	1090	692
Mixed venous O_2_ sat (%)		29.6	57.1	53.4	55.2	52.8	54.3
Hemolysis labs							
Platelets (10^3^/uL)	127	105	88	65	39	50	106
Hb (g/dL)	12.1	12.8	11.2	9.8	9.2	9.4	9.3
LDH (U/L)	N/A	N/A	2543	4685	6150	2396	N/A
Thyroid labs							
TSH (u/mL)	0.01				0.02		
Free T3 (pg/mL)	32				3.1		3.5
FREE T4 (ng/mL)	7.7				5.6		2.1

^a^ Prior to impella placement.

^b^ Post‐Bi‐Pella placement.

^c^ Prior to RP removal.

^d^ Prior to CP removal.

## 2 CASE SERIES

### 2.1 Management/Interventions

**FIGURE 1:**
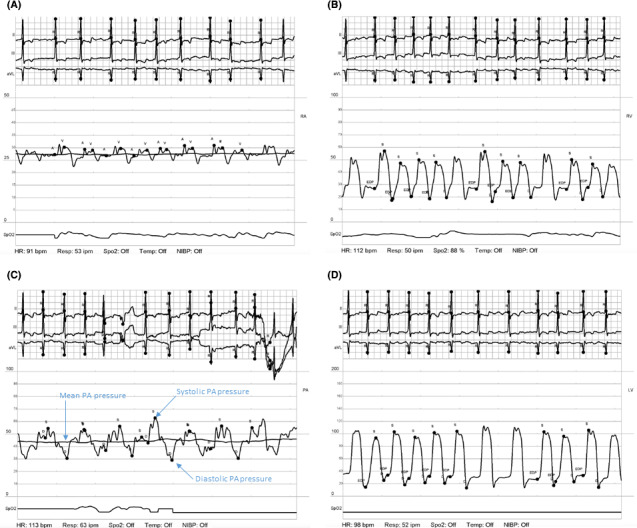
Initial right heart hemodynamics prior to impella insertion: Right atrial waveform (A) demonstrating significantly elevated RAP. B, RV waveform demonstrating an elevated RVP. C, PA pressures demonstrating an elevated PA pressures and a narrowed pulse pressure consistent with severely reduced RV function. D, Initial LV waveform showing a reduced LV pressure consistent with cardiogenic shock

**FIGURE 2:**
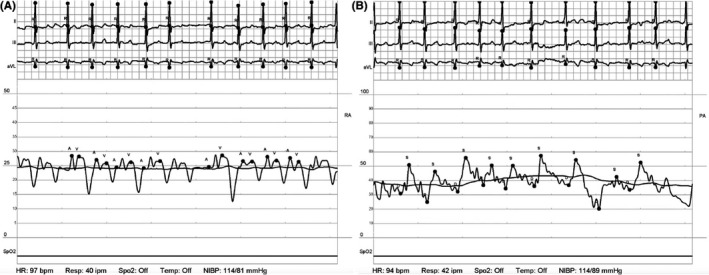
Right heart hemodynamics on Impella CP. Repeat waveforms of the right atrium (A) and pulmonary artery (B) after impella CP placement. The elevated right‐sided filling pressures and a narrow PA pulse pressure indicate minimal improvement in RV function after 30 min of LV unloading, consistent with intrinsic RV failure

A right heart catheterization was performed (Figure 1, Table 2). The initial pulmonary artery pulsatility index (PAPi) was 0.535, and his cardiac power output was 0.47 consistent with severe biventricular failure. An impella CP was placed via the right femoral artery. He was monitored in the cath lab for 30 minutes, and repeat right heart catheterization was performed (Figure 2):

**TABLE 2:** Hemodynamic trend in cardiac catheterization lab

Hemodynamic measurement	Initial cardiac hemodynamics	Post‐impella CP placement	Post‐Bi‐Pella placement
Right atrial pressure (mm Hg) (normal: 0‐5)	28	26	22
Right ventricular pressure (mm Hg) (normal: 25/5)	50/19	N/A	39/10
Pulmonary artery pressure (mm Hg) (normal: 25/10)	54/39 (45)	51/32 (40)	45/22 (33)
Pulmonary capillary wedge Pressure (mm Hg) (normal <12)	27	25	25
Pulmonary artery saturation (%) (normal: 50‐70)	29.6	57.1	N/A
Left ventricular pressure (mm Hg) (LVEDP) (normal <15)	104/20 (28)	N/A	N/A
Aortic pressure (mm Hg) (MAP)	96/86 (83)	100/78 (86)	101/72 (82)
Aortic saturation (%)	86.9	86.9	N/A
Calculated values			
Stroke volume (mL/beat) (normal: 60‐100)	28.1	47.8	N/A
RV stroke work index (normal: 8‐12)	3.19	4.48	N/A
RA/PCWP (0.3‐0.6)	1.03	1.04	0.88
Cardiac output (L/min) (normal: 4‐8)	2.8	5.3	N/A
Cardiac index (L/(min^a^m^2^) (normal 2.5‐4)	1.4	2.6	N/A
Systemic vascular resistance (dynes) (normal: 700‐1600)	1571	905	N/A
Pulmonary vascular resistance (woods units) (normal: 0.25‐1.6)	6.4	2.82^a^	N/A

^a^ Assume LAP = PCWP

**FIGURE 3:**
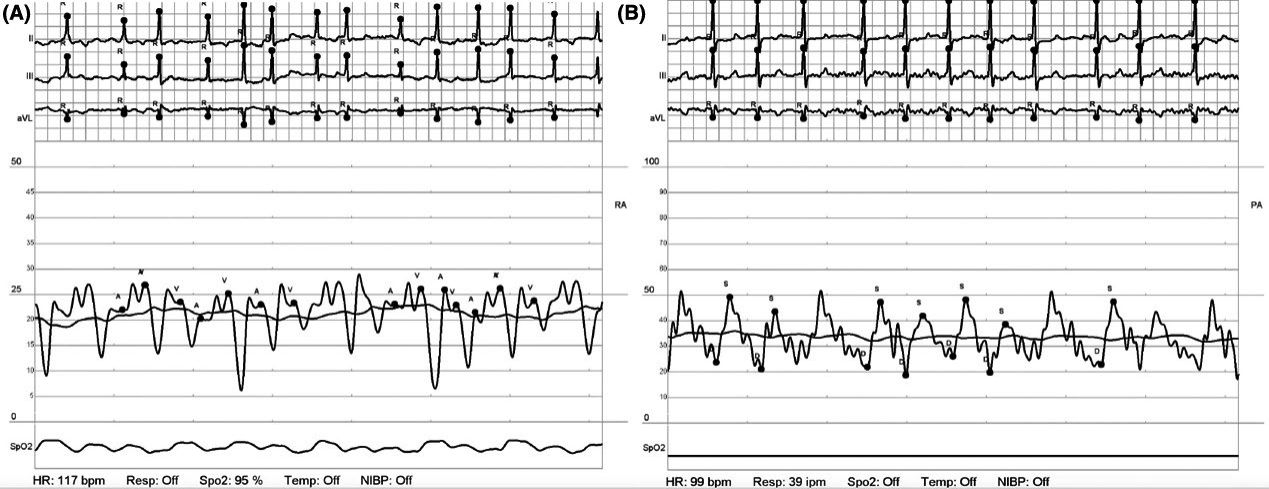
RA and PA waveforms following placement of RP impella. Repeat waveforms of the right atrium (A) and pulmonary artery (B) demonstrate widening of the PA pulse pressure with brisk down‐stroke and improved RA pressures consistent with improved RV output

Repeat PAPi was 0.73 demonstrating minimal improvement in RV function with LV offloading. The RA:PCWP was 1.04 which was unchanged from the initial measurement prior to impella CP placement, and the RV stroke work index (RVSWI) improved modestly from 3.2 to 4.5, but was still significantly reduced, indicative of severe RV dysfunction. At that point, an RP impella was placed and repeat hemodynamics showed improved biventricular function (Figure 3, Table 2) with an improved PAPi of 1.04 and an RA: PCWP of 0.88. He was then transferred to the CCU for further management.

In the CCU, he was placed on PTU, hydrocortisone, cholestyramine, lugol's solution, and propranolol for management of his hyperthyroidism and rate control.

Over the course of 3 days his lactate, renal and hepatic function drastically improved. He developed mild hemolysis, hematuria, and thrombocytopenia, all of which improved following device weaning (Table 1).

On hospital day 3, echocardiograhy showed modest improvement in the right ventricular function (TAPSE improved from 1.02 cm to 1.73 cm and RVOT TVI improved from 4.75 cm to 13.4 cm) prompting removal of the RP impella. He was aggressively diuresed, and on hospital day 4, the impella CP was weaned off and removed.

Diuresis and uptitration of guideline‐directed medical therapy for heart failure were continued over several days. On hospital day 8, he underwent successful TEE/DCCV. Prior to discharge, repeat echocardiogram showed marked improvement with an ejection fraction of 35% and normal right ventricular function by TAPSE. On discharge, he was taking metoprolol succinate, lisinopril, furosemide, apixaban, and methimazole.

Clinical Follow‐up: 6 months later, he was asymptomatic and had an echocardiogram that showed normalized cardiac function.

## 3 DISCUSSION

In hyperthyroidism, the increased serum concentration of T3 up‐regulates several cardiac‐specific genes enhancing contractility, improving cardiac relaxation, lowering SVR, increasing blood volume, and elevating baseline heart rate.^1^ In the setting of prolonged, severe hyperthyroidism, sustained tachycardia impairs left ventricular contractility and increases atrial‐filling pressures leading to heart failure. Ultimately, an untreated high‐output state may lead to ventricular dilatation and persistent tachycardia resulting in cardiogenic shock.^2^ 6% of patients with thyrotoxicosis develop symptoms of heart failure, but <1% develop a dilated cardiomyopathy and impaired systolic function.^3^ Of these patients with impaired systolic function, cardiogenic shock is rare, but with mortality rates that approach 30%.^4^

The above case illustrates how rapid recognition of the severity of illness accompanied by aggressive management with mechanical circulatory support can result in a good outcome and avoid serious comorbidities. While there are no prospective studies that determine treatment in thyroid storm, the current standard of care based on expert opinion consists of administering a beta blocker for control of adrenergic surge, use of a thionamide (in this case PTU) to block new synthesis, glucocorticoids to reduce T4‐T3 conversion, and an iodine agent to block release of thyroid hormone. Given the relative frequency of atrial fibrillation but relatively uncommon complication of cardiogenic shock in this population, it is not uncommon to initiate beta blockade without first assessing cardiac function. Furthermore, on presentation our patient appeared deceptively stable, which is likely due to his young age and ability to significantly increase his SVR despite a thyrotoxic state. This likely resulted in a false sense of security that beta blockade initiation would be well tolerated. When his heart rate dropped, his poor cardiac output led to rapid clinical decline and laboratory evidence of multi‐organ hypo‐perfusion. While it is not possible to delineate whether his ventricular dysfunction was due to prolonged tachyarrhythmia or thyrotoxicosis, this difference did not affect management as he required support while the underlying disorder was treated. The necessity of beta blockade to treat both his adrenergic surge and atrial fibrillation coupled with his severely reduced cardiac function illustrates the management dilemma in our patient and the importance of using mechanical support.

Given his deteriorating clinical status and limited medical options, the invasive hemodynamics played an integral role in determining the step‐wise approach to mechanical support. In the laboratory, there was clear evidence of severely impaired left ventricular function by CPO. Several hemodynamic measurements including PAPi, RA:PCWP ratio, and RVSWI were indicative of severe right ventricular dysfunction (Table 2). Previous studies to evaluate the most effective management of biventricular failure have yielded mixed results.^5^ Given the limited options for Medical therapy, the use of acute mechanical circulatory support has grown as Impella, TandemHeart, and VA‐ECMO have been used with increased frequency over the last 15 years.^6^

The decision to place an impella CP was made based on what we identified as a reversible cause of cardiogenic shock. The use of impella over ECMO was based on the concept of ventricular unloading to allow the ventricle time to recover as the underlying cause of heart failure was treated. Following placement of the impella CP, the patient was monitored in the cath lab to see if the right ventricular function improved with offloading of the left ventricle. This resulted in a slight improvement of his PAPi and RVSWI; however, both were still severely reduced and his RA:PCWP ratio was unchanged and severely elevated. Prior studies have indicated that a reduced RVSWI is an independent predictor for biventricular support requirement in patient undergoing LVAD placement^7^ and that an increased RA:PCWP ratio is associated with reduced RV function and adverse outcomes in advanced heart failure.^8^ Given these previous data, combined with hemodynamic findings and minimal clinical improvement, we determined that RV support, in addition to LV support, was necessary to allow for treatment of his underlying disorder. Prior evidence indicates that biventricular impella (Bi‐Pella) is a feasible approach that improves cardiac output and may be associated with improved outcomes in patients with biventricular failure.^9^ Thus following the Recover Right Trial, an RP impella was placed^10^ which showed an improvement in his PAPi and RA:PCWP ratio (Table 2) and more importantly a significant improvement in his mental status.

What followed was rapid improvement in his overall clinical status. Multi‐organ failure was reversed within 48 hours of mechanical support, and the need for other advanced supportive care was avoided. By 48 hours, there was significant improvement in biventricular function. With resolution of clinical cardiogenic shock and evidence of hemolysis (Table 1), the RP impella was discontinued. Following this, the hemolysis improved and the impella CP was discontinued shortly thereafter.

This case illustrates that, despite contrasting evidence, the use of Bi‐Pella may have a significant impact on mortality in patients with acute, reversible causes of cardiogenic shock. In this setting, the use of the device both supported the patient through shock and allowed the continued use of beta blocker therapy possibly resulting in a quicker recovery. The short‐term use of the support device likely limited our device‐related complications to mild hemolysis and thrombocytopenia. This leads us to believe that Bi‐Pella has a “sweet spot” for duration of support where a mortality benefit can be gleaned. Further studies should be done to determine the balance between duration of support and degree of ventricular recovery that will have the greatest benefit in patient care.

One limitation in our case is that for our cardiac output we utilized the Fick equation with nomogram‐derived estimates of O_2_ consumption. In a thyrotoxic state, this may underestimate true O_2_ consumption and lead to an underestimation of cardiac output and an overestimation of SVR. However, the patient had severe tricuspid regurgitation by echocardiogram, and in his clinically low‐flow state, thermodilution likely would yield similar limitations. Second, given his degree of illness, full hemodynamics were not performed at every stage, but only as necessary to direct management.

## 4 CONCLUSION

Our case demonstrates that rapid diagnosis and aggressive mechanical intervention in acute, reversible cardiogenic shock with Bi‐Pella can improve clinical outcomes without significant comorbidity. Further studies are needed to determine the ideal balance between ventricular offloading and duration of support so as to derive a mortality benefit with this device.

## CONFLICT OF INTEREST

The authors, Evan Caruso and Elias Iliadis, have no conflicts of interest to disclose with respect to the topics and management presented in this article.

## ETHICAL APPROVAL

This study was performed with all appropriate ethical considerations with consent from all providers and patients included in the study.

## AUTHOR CONTRIBUTIONS

EC: collected background data, involved in analysis of hemodynamics, and is a primary author of manuscript development. EI: is a primary investigator, physician who managed patient and performed cardiac catheterization, and provided significant authorship in the development of manuscript.

## Data Availability

All data included in this study are accurate in its entirety to the best knowledge of the investigators. All data are available for review by request of any interested entity.
